# Phenotypic and Genotypic Characterization of Biofilm Forming Capabilities in Non-O157 Shiga Toxin-Producing *Escherichia coli* Strains

**DOI:** 10.1371/journal.pone.0084863

**Published:** 2013-12-27

**Authors:** Chin-Yi Chen, Christopher S. Hofmann, Bryan J. Cottrell, Terence P. Strobaugh Jr, George C. Paoli, Ly-Huong Nguyen, Xianghe Yan, Gaylen A. Uhlich

**Affiliations:** Molecular Characterization of Foodborne Pathogens Research Unit, Eastern Regional Research Center, Agricultural Research Service, United States Department of Agriculture, Wyndmoor, Pennsylvania, United States of America; University of Birmingham, United Kingdom

## Abstract

The biofilm life style helps bacteria resist oxidative stress, desiccation, antibiotic treatment, and starvation. Biofilm formation involves a complex regulatory gene network controlled by various environmental signals. It was previously shown that prophage insertions in *mlrA* and heterogeneous mutations in *rpoS* constituted major obstacles limiting biofilm formation and the expression of extracellular curli fibers in strains of *Escherichia coli* serotype O157:H7. The purpose of this study was to test strains from other important serotypes of Shiga toxin-producing *E. coli* (STEC) (O26, O45, O103, O111, O113, O121, and O145) for similar regulatory restrictions. In a small but diverse collection of biofilm-forming and non-forming strains, *mlrA* prophage insertions were identified in only 4 of the 19 strains (serotypes O103, O113, and O145). Only the STEC O103 and O113 strains could be complemented by a trans-copy of *mlrA* to restore curli production and Congo red (CR) dye affinity. RpoS mutations were found in 5 strains (4 serotypes), each with low CR affinity, and the defects were moderately restored by a wild-type copy of *rpoS* in 2 of the 3 strains attempted. Fourteen strains in this study showed no or weak biofilm formation, of which 9 could be explained by prophage insertions or *rpoS* mutations. However, each of the remaining five biofilm-deficient strains, as well as the two O145 strains that could not be complemented by *mlrA*, showed complete or nearly complete lack of motility. This study indicates that *mlrA* prophage insertions and *rpoS* mutations do limit biofilm and curli expression in the non-serotype O157:H7 STEC but prophage insertions may not be as common as in serotype O157:H7 strains. The results also suggest that lack of motility provides a third major factor limiting biofilm formation in the non-O157:H7 STEC. Understanding biofilm regulatory mechanisms will prove beneficial in reducing pathogen survival and enhancing food safety.

## Introduction

Shiga toxin-producing *Escherichia coli* (STEC) has emerged in the last three decades to become one of the most important causes of food-associated illnesses in the United States and abroad [1-3]. It has been estimated that serotype O157:H7 is isolated from about one-half of the cases of STEC-induced diarrheal diseases in North America, while non-O157:H7 serotypes comprise the remaining isolates [1]. Of the non-O157:H7 STEC submitted from state public health laboratories for confirmation by the Centers for Disease Control and Prevention (CDC), six O-serogroups (O26, O45, O103, O111, O121, O145) accounted for 71% of all isolates in the period from 1983-2002 [1]. In recognition of the safety concerns and the higher incidence associated with these 6 O-serogroups, the United States Department of Agriculture, Food Safety Inspection Service (FSIS) declared STEC serogroups O26, O45, O103, O111, O121, and O145 to be adulterants in raw non-intact beef products and product components in 2011 and initiated a testing program for these 6 STEC in beef trimmings in June 2012 [4]. Recent studies have identified O113:H21 as an additional important STEC serotype, being the most frequent serotype isolated from ground beef samples [5] and belonging to the same seropathotype as serotype O121:NM in the classification scheme conceived by Karmali et al. [6].

The importance of biofilms in human disease pathogenesis and in the food industry has been well established [7,8]. In *E. coli*, biofilm formation in the majority of strains is dependent on the expression of the curli fimbriae (encoded by *csgBA*) and synthesis of polysaccharides such as cellulose [9]. The expression of curli and cellulose was found to be highly variable at 28°C and 37°C in a study of commensal *E. coli* isolated from the human gastrointestinal tract [10]. Expression of both curli and cellulose is dependent on the DNA-binding transcription factor CsgD [11]. The *csgD* gene is expressed from a relatively weak promoter located in the intervening region between the divergently transcribed *csgDEFG* and *csgBAC* operons, and relies on a complex network of regulatory factors, both activators and suppressors, to coordinate transcription under a variety of environmental conditions [11-15]. CsgD expression in *Salmonella enterica* serovar Typhimurium and *E. coli* had been shown to be affected by environmental conditions such as temperature, pH, oxygen concentration, osmolarity, and various nutrients [11]. Transcription of *csgD* predominately utilizes the stationary phase sigma factor RpoS, which activates *ydaM* and *mlrA* in a temporal fashion before cooperating with both to activate *csgD* transcription [16]. Both MlrA and YdaM are essential for *csgD* expression [15,16].

Although there have been numerous studies focused on the mechanisms and regulation of biofilm formation in both non-pathogenic and pathogenic *E. coli*, there is a shortage of similar data on STEC strains, especially the clinically important non-O157:H7 serogroups [17,18]. In a previous study using 55 clinical isolates of *E. coli* serotype O157:H7, we determined that only 4 of the strains showed a weak affinity for the curli-binding dye, Congo red (CR), when cultured on CRI agar at 25°C [19]. None of the strains could generate significant biofilm on polystyrene at 25°C when cultured in LB broth without salt (LB-NS) [19]. Moreover, we determined that these deficiencies were the result of either a lack of *mlrA* expression due to prophage insertions in the proximal coding region of *mlrA* (53/55 strains), or attenuating or inactivating lesions in RpoS (40/55 strains). Approximately 70% (38/55) of the O157:H7 strains carried a prophage in *mlrA* and possessed an RpoS mutation. Apparently, if strains of serotype O157:H7 are to assume a curli and biofilm expressing phenotype, they must make regulatory adjustments or accumulate mutations that overcome these barriers. It is unclear if other serotypes of pathogenic *E. coli* are subject to the same regulatory constraints. In two surveys of non-O157:H7 STEC strains, prophage insertions in *mlrA* (*yehV*) were found at a lower frequency in the non-O157:H7 STEC strains than in serotype O157:H7 strains suggesting that disruption of *mlrA* may not be an important barrier to curli expression in the non-O157:H7 STEC serotypes [20,21].

In this study we assembled 19 curli-producing and non-producing strains from 7 important, non-O157:H7 O-serogroups to determine whether deficiencies in *mlrA* and *rpoS* impose major barriers to curli production and biofilm formation as in clinical isolates of serotype O157:H7. 

## Materials and Methods

### Bacterial strains, growth conditions, plasmids and primers

Two or more strains from each of the seven *E. coli* O-serogroups O26, O45, O103, O111, O113, O121, and O145 were collected and used in this study (Table 1). The selection criterion was based on the varying degrees of biofilm-forming capabilities assessed by CR dye affinity following 48-h growth on Congo red indicator (CRI) agar at 25°C [25]. Serotype O157:H7 strain ATCC 43895 was used as a negative control, and strain 43894OR, a constitutive curli-producing strain [25], was used as a positive control for curli isolation and for CR affinity. Overnight cultures were grown in Luria-Bertani (LB) broth at 37°C or 30°C unless otherwise specified. Plasmids used for complementation experiments (pUC19::*mlrA* and pTOPO::*rpoS*) were described previously [19]. Complementation plasmids were introduced into all the STEC strains by electroporation using an *E. coli* Pulser (Bio-Rad, Hercules, CA).

**Table 1:** List of the STEC strains used in this study.

**Strain**	**Serotype**	*stx_1_* ^a^	*stx*_2_^a^	Resistance^b^	*csgD* promoter^c^	Source^d^	Reference
FCL1	O103:H2	**+**	**-**		pC	Vegetable	This study
DA-33	O103:H2	**+**	**-**		pB/G	MSU	[22]
SJ10	O103:H2	**+**	**-**		pC	CDC	[22]
SJ14	O111:H8	**+**	**+**		pB	CDC	[22]
98-8338	O111:NM	**+**	**-**	AmpKan	pB	PHAC	[22]
SJ13	O111:NM	**+**	**+**	AmpKan	pB	CDC	[22]
SJ15	O113: ?	**+**	**+**		pB	CDC	[22]
SJ29	O113:H21	**-**	**+**		pB	CDC	[22]
04-1450	O113:H21	**-**	**+**		pB	PHAC	[23]
DEC10	O26:H11	**+**	**-**		pB	MSU	[22]
05-6544	O26:H11	**+**	**-**		pB	PHAC	[22]
05-6545	O45:H2	**+**	**-**		pC	PHAC	[22]
96-3285	O45:H2	**+**	**-**	AmpKanTet	pC	PHAC	[22]
SJ7	O45:H2	**+**	**-**		pC	CDC	[22]
SJ16	O121:H19	**-**	**+**		pD	CDC	[22]
SJ18	O121:H19	**+**	**+**		pD	CDC	[22]
03-4064	O121:NM	**-**	**+**	Amp	pD	PHAC	[22]
SJ24	O145:NM	**-**	**+**		pE	CDC	[22]
E59	O145:H28	**-**	**-**		pF	ARS	[24]

^a^ *stx*_*1*_ and *stx*_*2*_ PCR results are shown in Figure S1.

^b^ Amp, ampicillin; Kan, kanamycin; Tet, tetracycline.

^c^ GenBank Accession numbers of the *csgD* promoters are KF680304-KF680323, respectively. pB/G has three additional nucleotide changes in *csgD* CDS compared to those of pB promoters.

^d^ Vegetable, original strain designation FCL1-2556 isolated from vegetables in the field, provided by Dr. Cristobal Chaidez at Centro de Investigación en Alimentación y Desarrollo, Culiacan, Mexico; ARS, Agricultural Research Service, Eastern Regional Research Center; CDC, US Centers for Disease Control and Prevention, Atlanta, Georgia, USA; PHAC, Public Health Agency of Canada, Winnipeg, Manitoba, Canada; MSU, Michigan State University Department of Microbiology and Molecular Genetics STEC Center, East Lansing, Michigan, USA

### DNA isolation, PCR and sequencing

DNA was isolated from 18-24 h cultures using the DNeasy Blood and Tissue Kit (Qiagen, Valencia, CA). Primers used in this study are shown in Table 2. O-serogroup and the status of the *mlrA* gene were investigated using previously described primers in a multiplex format [19,20,27]. PCR verification for *stx*_*1*_ and *stx*_*2*_ genes were performed using published primers [26]. All PCR assays (25 µl) were set up using the Qiagen Multiplex PCR Master Mix (Qiagen), 0.5X Q-Solution, 1.5 µl template DNA, and primers at a final concentration of 0.5 µM each. Cycling conditions were as recommended by the manufacturer: 95°C for 15 min, followed by 35 cycles of 30 s at 94°C/ 90 s at 52°C for stx1-comF/R and GK5/GK6, or 57°C for all others/ 90 s at 72°C, and then 10 min at 72°C. The *mlrA* and *rpoS* genes, including flanking regions, were amplified using the primer pairs MlrA-F/MlrA-R and RpoS-F/RpoS-R, respectively. The *csgBA* genes and the intergenic region between *csgB* and *csgD* were amplified using CsgB-PF/CsgA-PR and CsgDup2/CsgDdown1, respectively. Amplified products were sequenced using custom primers [19], the Big Dye Terminator (v3.1) and ABI 3730 DNA Analyzer (Life Technologies) at our core facility. Sequences were assembled using Geneious (v5.6; Biomatters) or Sequencher (v5.1; Gene Code). Sequence annotation and analyses were performed using Geneious (v5.6). Sequences were deposited in NCBI GenBank under accession numbers KF662824-KF662843 for *mlrA*, KF662844-KF662863 for *rpoS*, KF662816-KF662823 for *csgBA* (8 strains), and KF680304-KF680323 for *csgD* promoter regions.

**Table 2:** Primers and plasmids used in this study.

Primer or plasmid	Sequence (5’ → 3’) or description	Reference	Purpose
A (yehVRJfor)	AAGTGGCGTTGCTTTGTGAT	[20]	Multiplex *mlrA* PCR
B (yehVLJrev)	AACAGATGTGTGGTGAGTGTCTG	[20]	Multiplex *mlrA* PCR
E (yehVRJrev)	GATGCACAATAGGCACTAGGC	[20]	Multiplex *mlrA* PCR
F (yehVLJfor)	CACCGGAAGGACAATTCATC	[20]	Multiplex *mlrA* PCR
Stx1com-F	YAGTTGAGGGGGGTAAAATG	[26]	*stx_1_* PCR
Stx1com-R	CGRAAAATAACYTCGCTGAATC	[26]	*stx_1_* PCR
LP43	ATCCTATTCCCGGGAGTTTACG	[26]	*stx*_*2*_ PCR
LP44	GCGTCATCGTATACACAGGAGC	[26]	*stx*_*2*_ PCR
GK5	ATGAAGAAGATGTTTATG	[26]	*stx*_*2*_ PCR
GK6	TCAGTCATTATTAAACTG	[26]	*stx*_*2*_ PCR
O26F	CAATGGGCGGAAATTTTAGA	[27]	O serotype PCR
O26R	ATAATTTTCTCTGCCGTCGC	[27]	O serotype PCR
O45F	TGCAGTAACCTGCACGGGCG	[27]	O serotype PCR
O45R	AGCAGGCACAACAGCCACTACT	[27]	O serotype PCR
O103F	TTGGAGCGTTAACTGGACCT	[27]	O serotype PCR
O103R	GCTCCCGAGCACGTATAAAG	[27]	O serotype PCR
O111F	TGTTTCTTCGATGTTGCGAG	[27]	O serotype PCR
O111R	GCAAGGGACATAAGAAGCCA	[27]	O serotype PCR
O113F	TGCCATAATTCAGAGGGTGAC	[27]	O serotype PCR
O113R	AACAAAGCTAATTGTGGCCG	[27]	O serotype PCR
O121F	TCCAACAATTGGTCGTGAAA	[27]	O serotype PCR
O121R	AGAAAGTGTGAAATGCCCGT	[27]	O serotype PCR
O145F	TTCATTGTTTTGCTTGCTCG	[27]	O serotype PCR
O145R	GGCAAGCTTTGGAAATGAAA	[27]	O serotype PCR
RpoS-F	TATCGCCTGGATTACTGGCAAC	[19]	*rpoS* amplification/sequence
RpoS-R	TAGGACGCTGACGTGTCTTATC	[19]	*rpoS* amplification/sequence
MlrA-F	ACATACCCGCAAACCACACTTC	[19]	*mlrA* amplification/sequence
MlrA-R	AGCTATGCGCATAATGCACTCC	[19]	*mlrA* amplification/sequence
CsgDup2	ACTACCTTCTTGCGCAACAACC	This study	*csgD* promoter activity
CsgDdown1	GAAGGATCCAGGATAAGCTTTTTATCCGCTTCC	This study	*csgD* promoter activity
CsgB-PF	TCCGCAGACATACTTTCCATCG	This study	*csgBA* amplification/sequence
CsgA-PR	ATTTGAAAGTGCGGCAAGGAGC	This study	*csgBA* amplification/sequence
CsgB-PR	AATTCGGGCCGCTGTTATTACC	This study	*csgBA* sequence
CsgA-PF	TGCAGAGACAGTCGCAAATGG	This study	*csgBA* sequence
pUC19::*mlrA*	mlrA + 224 bp upstream	[19]	Trans-complementation
pTOPO::*rpoS*	rpoS + 804 bp upstream	[19]	Trans-complementation

### Congo red dye affinity assay

CR binding was assessed by spotting 3 µl of overnight LB cultures on CRI plates and incubating for 48 h at 25°C. Plates were imaged using Epson Perfection 3200 Photo scanner and Epson Scan software (Professional mode) with a blue background at 600 dpi/48-bit color.

### Motility assay

Motility was measured using the soft agar swimming assay modified from previously described [28]. Briefly, bacterial strains were grown in LB-NS for 48 h at 25°C without shaking.  Soft agar plates (1% tryptone, 0.5% NaCl and 0.3% agar) were inoculated by stabbing the center of the plate with a toothpick wetted with the broth cultures.  The diameters of the halos grown at 25°C were measured after 24 h. 

### Catalase tests

Strains were screened for catalase activity by placing a small loop-full (~1 µl) of bacteria collected from an 18-24-h colony onto a glass slide and exposing the bacteria to a drop of 30% H_2_O_2_. Results were graded by the robustness of bubbling, from “- (very little or no bubble formation)” to “+++ (immediate, strong bubble formation)”. 

### Biofilm (crystal violet-binding) assays

Starter cultures were grown in LB-NS at 37°C for 18 h. Cultures were diluted 1:100 in fresh LB-NS, and 100-µl samples of each strain were dispensed into 6 wells of a 96-well plate (TPP, Trasadingen, Switzerland). The plates with samples were incubated at 25°C for 48 h in a sealed humidity chamber to prevent loss of sample volume. Cultures were removed and the presence of biofilm/pellicles was visually inspected. The wells were then washed three times with water using a Nunc-Immuno Wash 8 plate washer (Nalge Nunc International). Biofilm was detected by addition of 200 µl of a 0.1% crystal violet (CV) solution per well for 30 min, followed by three water washes and de-staining with 200 µl of 95% ethanol for 30 min. The absorbance was read at 590 nm using Safire2 (TECAN).

### Curli enrichment and isolation

Curli fibers, which are resistant to SDS solubilization, were isolated and solubilized in formic acid using the procedures described previously [10,19]. Samples were loaded on an Any kD TGX gel (Bio-Rad) and resolved by electrophoresis in Tris-Glycine-SDS buffer (25 mM Tris, 192 mM Glycine, 0.1% w/v SDS, pH 8.3) (Bio-Rad). CsgA subunits were visualized with Bio-Safe Coomassie Stain G250 (Bio-Rad). 

### *csgD* promoter activity

Six representative *csgD* promoter types (Table 1) were amplified from strains ATCC 43895, SJ13, 05-6545, 03-4064, SJ24, and E59 using primers CsgDup2 and CsgDdown1 and cloned into *Sma*I-*Bam*HI digested pMLB1034 in *E. coli* EC100 strain to generate *lacZ* translational fusions. Cultures were grown in LB at 37°C and samples were collected at various time points and assayed for β-galactosidase activity [29]. Experiments were repeated three times, and one representative time course was shown.

## Results

**Figure 1:**
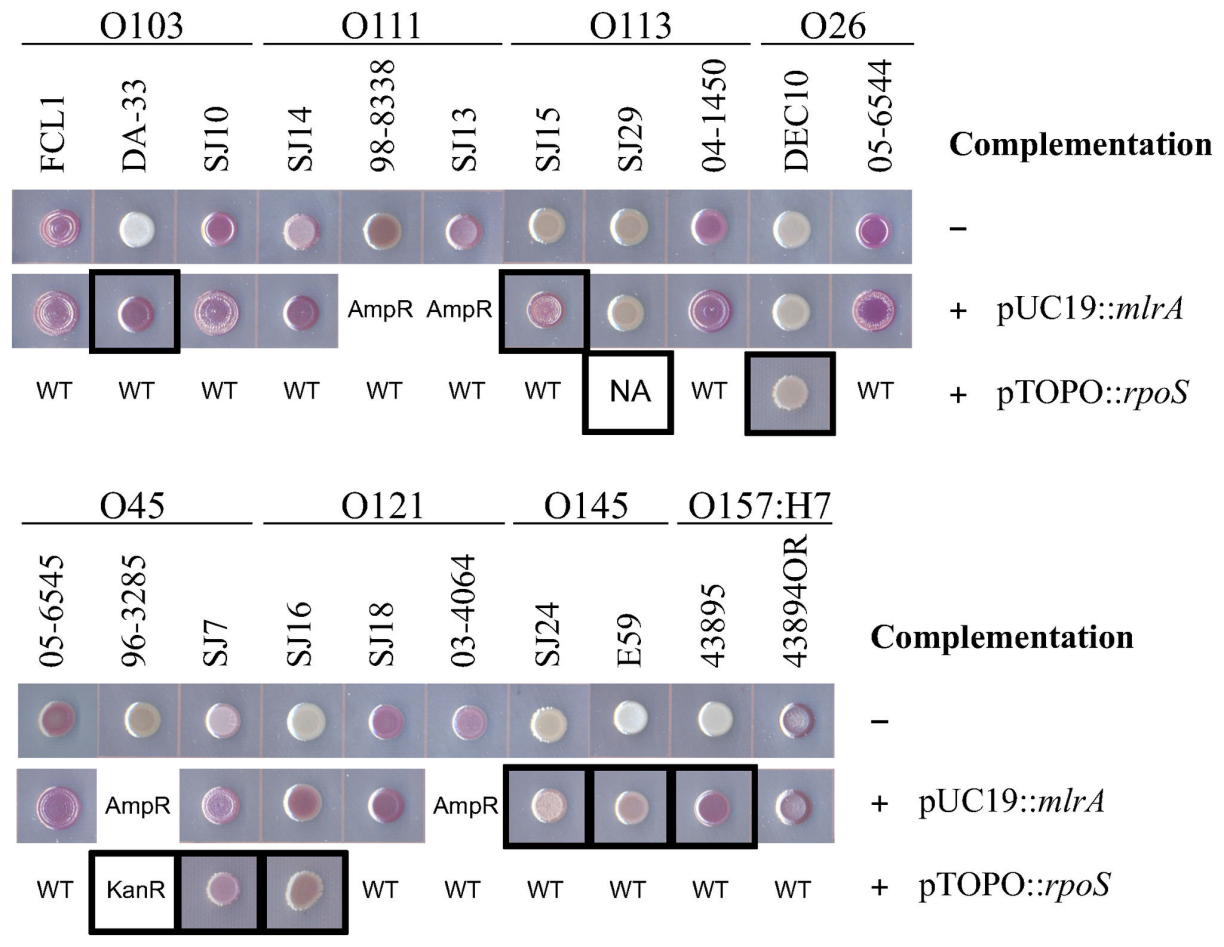
Congo red affinity of STEC strains with or without complementing plasmids pUC19::*mlrA* or pTOPO::*rpoS*. Strains carrying mutant *mlrA* or *rpoS* genes were marked with black boxes. AmpR/KanR: antibiotic resistance preventing the complementation experiments. WT: strain carries wild-type RpoS protein thus not complemented. NA: stable transformant not achievable.

Our previous study with 55 *E. coli* O157:H7 isolates showed that MlrA and RpoS functionality played a key role in determining the biofilm-forming capabilities of strains in that serotype; the majority of the strains carried a *stx*_*1*_ prophage insertion in *mlrA* and/or an *rpoS* mutation that disrupted the curli regulatory pathway, thus diminishing their ability to form biofilms as evidenced by CR and CV binding. In this study, nineteen isolates of the 7 important non-O157 STEC O-serogroups O26, O45, O103, O111, O113, O121, and O145 were selected based on their varied affinities for CR dye (Figure 1). The O serogroup and the status of the *stx*_*1*_, *stx*_*2*_ and *mlrA* genes are shown in Tables 1 and 3, and Figure S1. Results of the motility assay and catalase tests are shown in Table 3. Strain SJ15, previously thought to be O111:NM, tested negative in the latex agglutination assays developed for the “Top 6” non-O157 serotypes [22]. Molecular serotyping by PCR in this study showed that it belongs to serogroup O113, which was not included in the targeted O groups in the agglutination assays [22]. The serogroup result was confirmed by conventional serotyping at the *E. coli* Reference Center (University Park, PA; Chobi DebRoy, personal communication). SJ15 was found to be motile, however, the H-type was not determined.

**Table 3:** Biofilm, crystal violet-, Congo red-binding phenotypes and curli expression of STEC strains with or without complementation by pUC19::*mlrA*.

					**Parent strain**				**+pUC19::*mlrA*^*g*^**		
**Name**	**Motility^*a*^**	***mlrA^b^***	**Catalase**	**RpoS^*c*^**	**Biofilm (visible**)**^*d*^**	**CV^*e*^**	**CRI^*f*^**	**Curli**	**CV^*e*^**	**CRI^*f*^**	**Curli**
FCL1	**-**	WT (340bp)	**+++**	WT	**-** (1+/6)	1.12±0.67	R+++d	**++**	2.66±0.03	R+++d	**++**
DA-33	**+**	824bp, weak 702bp/340bp	**+++**	WT	**-**	0.13±0.02	W	**-**	1.61±0.09	R++(+)	**++**
SJ10	**+**	WT	**+++**	WT	**+++**	2.71±0.02	R++(+)	**++**	2.19±0.06	R+++d	**++**
SJ14	**±**	WT	**+++**	WT	**-**	0.29±0.07	P/R+	**++**	1.57±0.05	R++(+)	**++**
98-8338	**-**	WT	**+++**	WT	**-**	0.10±0.01	B	**(+)**	ND	ND	ND
SJ13	**-**	WT	**+++**	WT	**-** (1+/6)	0.22±0.07	R++d	**++**	ND	ND	ND
SJ15	**+**	824bp, weak 340bp	**+++**	WT	**-** (1+/6)	0.16±0.07	W	**-**	0.53±0.10	R+++d	**(+)**
SJ29	**+**	WT	**-**	FS_87_T_98_ (Ins)	**-** (1+/6)	0.11±0.02	W	**-**	0.08±0.001	W	**-**
04-1450	**+**	WT	**+++**	WT	**+++**	1.55±0.27	R+	**++**	1.96±0.54	R+++d	**++**
DEC10	**+**	WT	**+/-**	Q161P (Sub)	**++**	0.11±0.01	W	**-**	0.09±0.004	W	**-**
05-6544	**+**	WT	**+++**	WT	**+++**	2.14±0.39	R++(+)	**+**	2.15±0.12	R+++d	**++**
05-6545	**+**	WT	**+++**	WT	**+++**	2.05±0.28	B/R+	**-**	1.98±0.10	R+++d	**+**
96-3285	**+**	WT	**-**	FS_34_T_71_ (Del)	**++**	0.13±0.02	W	**-**	ND	ND	ND
SJ7	**+**	WT	**+**	R311P (Sub)	**++**	0.19±0.02	W/P+	**-**	0.54±0.20	P/R+d	**+**
SJ16	**+**	WT	**+**	FS_78_T_98_ (Ins)	**-** (1+/6)	0.11±0.01	W	**-**	0.09±0.003	B/R+	**-**
SJ18	**+**	WT	**+++**	WT	**+++**	1.13±0.34	R+	**+**	2.40±0.08	R++	**+**
03-4064	**-**	WT	**+++**	WT	**-/+**	0.13±0.01	P/R+	**-**	ND	ND	ND
SJ24	**-**	multiple bands	**++**	WT	**-** (1+/6)	0.11±0.02	W	**-**	0.89±0.09	W/P	**-**
E59	**-**	multiple bands	**+++**	WT	**+**	0.11±0.01	W	**-**	0.51±0.03	W/P	**-**
43895	**+**	824bp/702bp, weak 340bp	**+++**	WT	**-**	0.10±0.004	W	**-**	1.76±0.12	R++	**+**
43894OR	**+**	824bp/702bp, weak 340bp	**+++**	WT	**+++**	2.11±0.14	R+++d	**++**	0.89±0.09	R+++d	**++**

^a^ -, growth at, but not beyond, the inoculation site (< 3mm dia.); +, growth and spreading of colony with diameters from 44 to 90 mm; ±, growth and only slight spreading of colony (8 mm dia.) ^b^ Multiplex PCR results using primers A, B, E and F (see Figure S1) ^c^ Predicted RpoS amino-acid sequence: WT, wild-type (same as ATCC 43895; Accession # JX680258); FS, frame-shift; T, truncation. Type of non-synonymous nucleotide change was noted in parenthesis: Ins, insertion; Del, deletion; Sub, substitution. ^d^ The presence and absence of visible pellicles/biofilms was scored after media removal and before staining with CV. ^e^ Crystal violet (CV)-binding was measured by absorbance at 590 nm averaged from 6 wells ± standard deviation. OD_590_ values of the LB-NS media controls were 0.11±0.012 (for experiments of the parent strains) and 0.08±0.005 (for pUC19::*mlrA* complemented strains). ^f^ Color on CRI agar. R: red, W: white, P: pink, B: brown, d: dry-appearance. ^g^ pUC19::*mlrA* complementation. ND: not determined due to antibiotic resistance of the parent strain.

### The *mlrA* gene is intact in most isolates and without *stx_1_* prophage insertion

Most STEC strains (15 out of 19) used in this study carried an intact *mlrA* gene, although 13 strains were *stx*_*1*_+ by PCR, indicating that *stx*_*1*_-encoded bacteriophage may prefer a different chromosomal insertion site in certain non-O157:H7 STEC strains (Tables 1 and 3; Figure S1). DA-33 and SJ15 strains, both *stx*_*1*_-positive, produced a strong 824-bp F/B band with weak 340-bp A/B band by *mlrA* multiplex PCR, indicating that they likely carried prophage insertions in the proximal region of *mlrA* as described for strains of *E. coli* serotype O157:H7 [19] (Table 3; Figure S1). Both O145 strains SJ24 and E59 were PCR-negative for *stx*_*1*_ but produced weak A/B (340 bp), A/E (702 bp), and F/B (824 bp) bands indicative of the prophage insertion in *mlrA*, with additional larger molecular-weight bands suggestive of primer binding at other prophage sites in the genome. Sequences of the intact *mlrA* genes from the 15 STEC strains and MG1655 were compared. All the predicted MlrA proteins are extremely conserved, showing only 1 amino acid difference at residue 239; Five strains (2 O103 strains FCL1 and SJ10 and all 3 O45 strains) had a leucine (L239) and the rest of 11 strains had a phenylalanine (F239) (data not shown).

### RpoS gene and catalase activity

**Figure 2:**
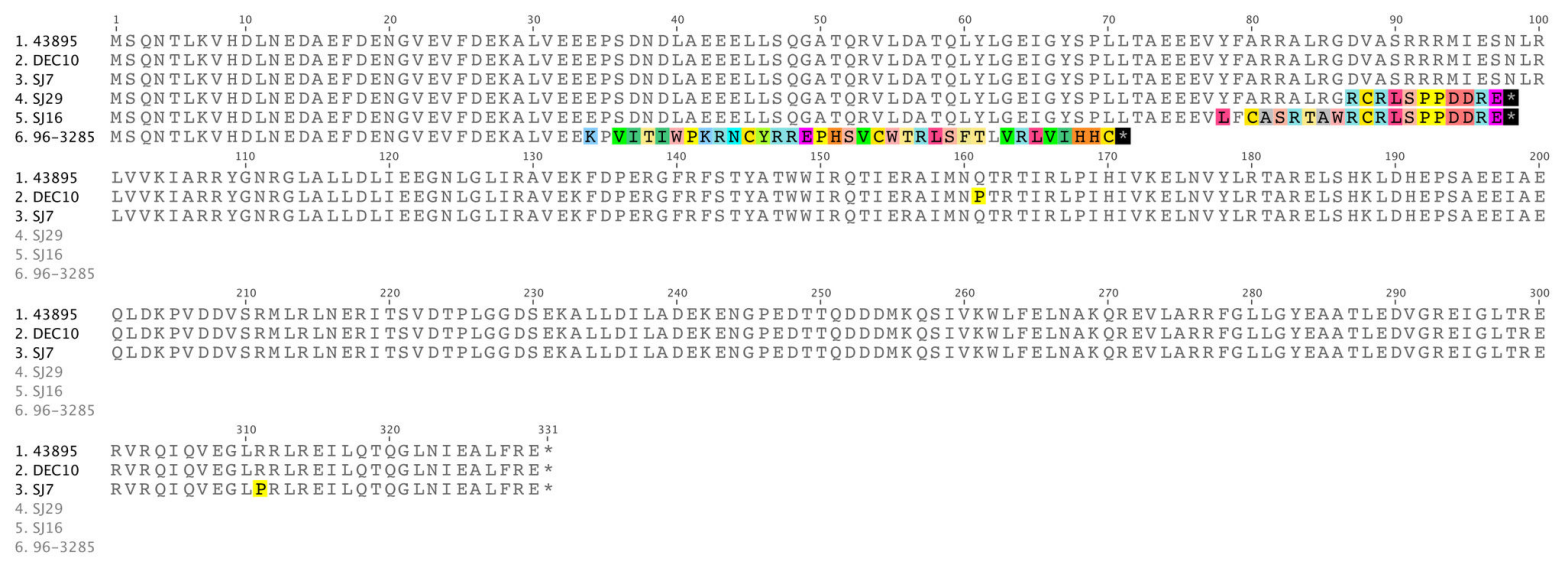
Protein alignment of RpoS. Mutant RpoS proteins from 5 STEC strains compared with the archetype strain ATCC 43895. Amino acids are listed by one-letter symbols.

DNA fragments containing the *rpoS* ORF and flanking regions were amplified and sequenced (GenBank Accession # KF662844-KF662863). Five different mutations in the *rpoS* coding region that resulted in amino acid alterations, compared to the RpoS of strain ATCC 43895, were identified in the 19 strains in this study, including 2 strains with an amino acid substitution, and 3 strains with a frame-shift due to single nucleotide insertion or deletion (Table 3 and Figure 2). None of these mutations were the same as those found in the O157:H7 strains PA1-PA52 examined previously [19]. All strains that produced mutant RpoS proteins showed decreased catalase activity using the hydrogen peroxide test (Table 3). 

### Congo red affinity and curli production

Results on CRI agar are shown in Figure 1 and listed in Table 3. Serotype O157:H7 strains ATCC 43895 (negative control) and 43894OR (positive control) were included for comparison. In contrast to O157 strains, non-O157 STEC strains showed a wider range of pink, brown, and red colors on CRI plates, likely due to the differences in their genetic backgrounds. All four strains that carried an interrupted *mlrA* appeared white on CRI. Four of the five *rpoS* mutant strains also appeared white, and one *rpoS* mutant strain (SJ7) showed a light pink color on CRI. 

**Figure 3:**
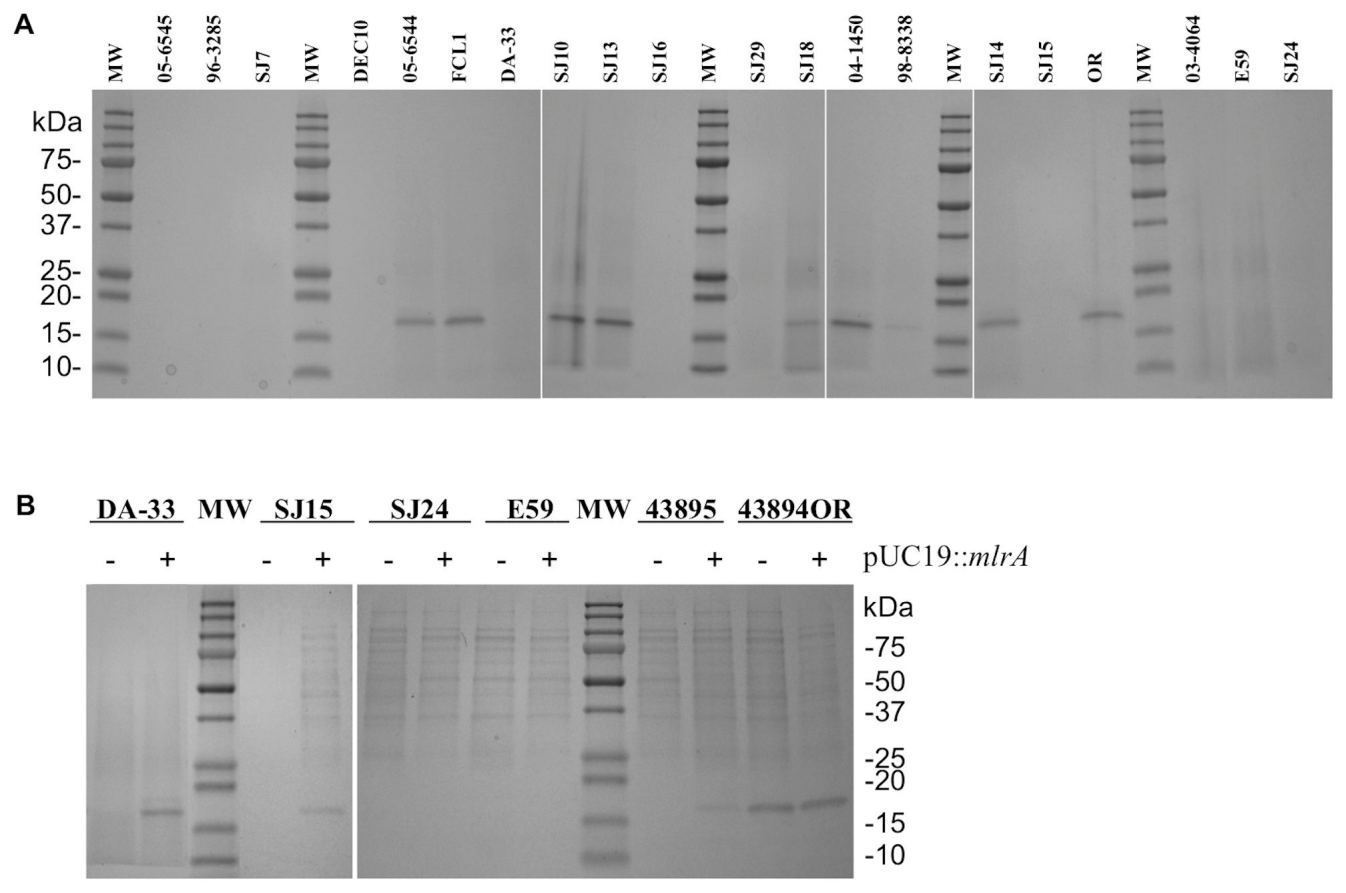
SDS-PAGE of CsgA (curli) extractions. (A) from parent strains and (B) from *mlrA* mutant strains complemented with pUC19::*mlrA* plasmid (+). Strain 43894OR was used as positive control. MW: Precision Plus dual color protein standards (Bio-Rad).

Production of curli/CsgA in the strains agreed with the CR affinity reasonably well (Figure 3 and Table 3). All 9 strains that were white on CRI did not produce curli. Most CR-binding isolates produced curli as evidenced by the CsgA bands on SDS-PAGE. However, four strains with weak to intermediate CR affinities showed little or no curli production: strains SJ7, 03-4064 and 05-6545 (light pink, pink/red+ and brown/red+, respectively) did not produce detectable levels of curli/CsgA, while strain 98-8338 (brown) produced only a trace amount. This is likely due to CR binding with other cell surface components (such as cellulose) in those strains (data not shown). 

### Biofilm formation (crystal violet-binding)

Biofilm formation was assayed in 96-well polystyrene plates using crystal violet (CV) dye binding (Table 3). Although most strains that showed intermediate to strong CR binding also bound CV strongly and formed visible biofilm, there were a few exceptions. Strains SJ13 and FCL1 bound CR strongly and had a dry appearance (Figure 1), but visual biofilm was observed in only 1 out of 6 replicate wells (1+/6) (Table 3). This variability may result from deficiencies in initial attachment due to their lack of motility, a stage of biofilm development prior to and thus independent of curli expression (Table 3). Lack of motility may also explain why strains SJ14 and 03-4064 showed little to no biofilm formation in spite of the fact that these strains bound CR to a modest degree, scoring pink/red+ (P/R+) on CRI. Strain 05-6545 was unique in that it was the only strain that produced abundant biofilm (CV OD_590_ = 2.05) and bound moderate amounts of CR but failed to express curli. No other curli-deficient strain generated enough biofilm to register CV OD_590_ values above 0.20. Western hybridization of the curli prep from strain 05-6545 with an *E. coli* CsgA specific antibody revealed a faint band after prolonged exposure, indicating that a low level of CsgA expression was present (data not shown). It is unclear whether the production of curli in 05-6545 was poor, or the curli fibers were structurally different from the norm thus eluding the purification procedure.

### Complementation by pUC19::*mlrA* or pTOPO::*rpoS* plasmids

Fifteen of the 19 strains were susceptible to ampicillin and were transformed with pUC19::*mlrA*. The pUC19::*mlrA* plasmid restored CR binding and curli production in two of the four strains carrying a disrupted *mlrA* gene (strains DA-33 and SJ15) (Figures 1 and 3, and Table 3). However, CR binding was not restored in the two O145 *mlrA* mutant strains (SJ24 and E59) or in strains SJ29 and DEC10 (the latter two strains carried *rpoS* mutations). Biofilm formation (evaluated by CV binding) was enhanced in all 4 pUC19::*mlrA* complemented *mlrA* mutant strains, and in only 1 of 5 *rpoS* mutant strains (SJ7). Since both SJ24 and E59 strains showed strong catalase activity, encoded wild-type RpoS proteins, and were not trans-complemented by *mlrA*, it must be assumed that their deficiencies in curli production and CR affinity resulted from defects in structural or regulatory genes other than *mlrA* or *rpoS*. 

Four of the 5 strains that carried a defective *rpoS* gene were susceptible to kanamycin (DEC10, SJ7, SJ16, and SJ29) and were transformed with pTOPO::*rpoS*. Strain 96-3285 was resistant to ampicillin, kanamycin and tetracycline (Table 1), thus it was excluded from the complementation study. Strain SJ29, although not resistant to kanamycin, was also excluded due to failure to stably maintain the pTOPO::*rpoS* plasmid. The catalase activity was successfully restored to near wild-type level by pTOPO::*rpoS* in strains DEC10, SJ7, and SJ16 (data not shown). SJ7 and SJ16 *rpoS-*complemented strains also showed slightly increased binding to CR, although not as strong as those complemented by pUC19::*mlrA* (Figure 1). CR-binding was unaffected by pTOPO::*rpoS* in strain DEC10, suggesting that this strain may have other defects in curli and/or CsgD expression in addition to the *rpoS* mutation. 

### CsgBA structural proteins

**Figure 4:**
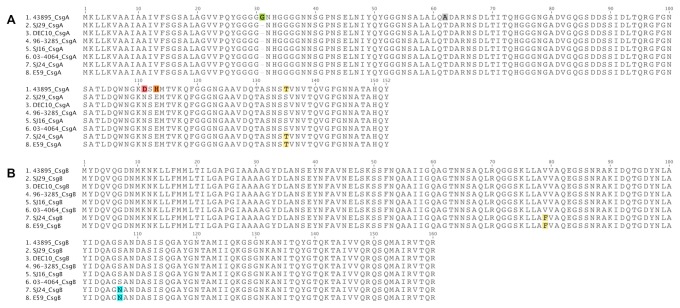
Protein alignments of CsgA (A) and CsgB (B) from selected STEC strains.

To rule out the possibility that the failure to produce curli was due to mutation or deletion in the curli structural genes, the *csgBA* coding regions were amplified and sequenced from the seven strains that failed to produce curli (SJ16, SJ29, DEC10, 96-3285, 03-4064, SJ24, E59) and 43895. There were over 40 nucleotide differences in the 1.16-kb region (pairwise identity 98.6%), but few resulted in amino acid changes (GenBank Accession # KF662816-KF662823). All seven non-O157 strains encoded full-length CsgB and CsgA. The deduced amino acid sequences of CsgA and CsgB were highly conserved (Figure 4). The CsgA proteins from the 7 non-O157 strains varied only by 1 amino acid at residue 134 (S or T). In addition to several amino acid substitutions, the most striking feature of the CsgA from O157:H7 strain 43895 was that it is one residue (glycine) longer in the glycine-rich region than those of the non-O157 strains due to a 3-bp insertion. However, this variation in the number of glycine residues was found in CsgA from other strains of *E. coli* in GenBank, thus it is not a rare occurrence (data not shown). The pI of CsgA protein from 43895 is 5.43, compared to 4.89 of the rest. All strains encoded identical CsgB proteins except for the two O145 strains (SJ24 and E59), which contained a phenylalanine rather than a valine at residue 79 and an asparagine rather than a serine at residue 107. These substitutions did not result in significant change in hydrophobicity, pI (9.58), or the predicted secondary structure of the CsgB protein. BLASTP results also showed that both amino acid changes were present in the CsgB from other strains of *E. coli* either singularly or in combination (data not shown). It is not clear whether these protein sequence variations could affect CR binding or biofilm formation, but it is certain that the lack of curli formation in these 7 strains was not due to truncations of the curli structural proteins. 

### Sequence and activity of the *csgD* promoters

**Figure 5:**
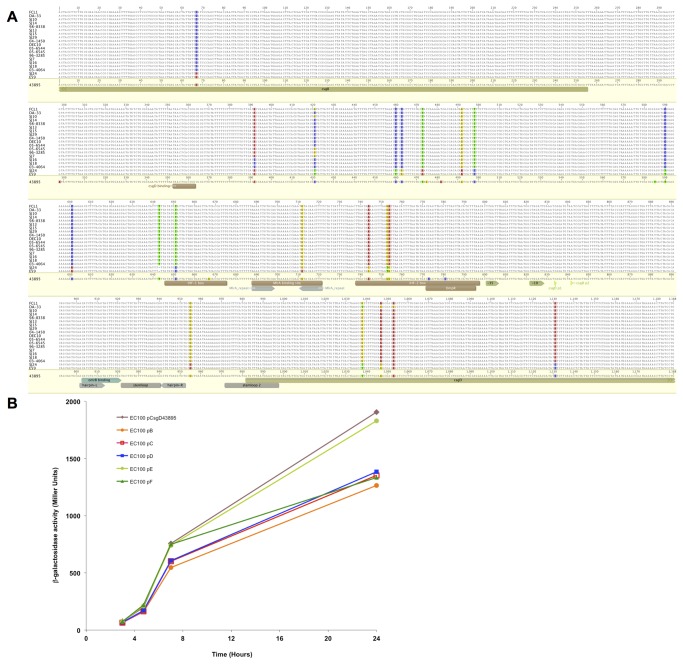
CsgD promoter sequence and activity. (A) Alignment of *csgD* promoters and (B) β-galactosidase activities (Miller Units) over time. A representative time course was presented.

As the extensive *csgD* promoter in *E. coli* contains numerous sequence-dependent binding sites for regulatory factors that modulate its expression, variations in the promoter sequence could affect promoter activity. The *csgD* promoter regions, including partial *csgB* and *csgD* coding sequences determined to be required for optimal expression of *csgD* in *S*. Typhimurium [30], was sequenced in each of the 19 non-O157 STEC strains for comparison with the sequence from *E. coli* O157:H7 strain ATCC 43895 (Figure 5). Five distinct promoter patterns (types B-F) were identified among the 19 strains, with type B being the predominant promoter type (Table 1). Promoter type B was found in each of the O26, O111, and O113 strains plus one O103 strain, DA-33, which carries 3 additional synonymous nucleotide changes in the *csgD* coding region (designated pB/G). Promoter type C was present in the three O45 strains and the 2 remaining O103 strains. Promoter type D was exclusively found in the three O121 strains. Types E and F, the promoters from the two O145 isolates, formed a separate cluster and differed from each other by only 1 bp. Thus there seemed to be some O-serogroup specificity for the *csgD* promoter sequences.

A *csgD::lacZ* fusion plasmid was constructed from each non-O157 promoter type B-F and from *E. coli* O157:H7 ATCC 43895, and transformed into the curli-expressing commercial K12 strain EC100. The cloned *csgD* coding regions were identical except for strains SJ24 and E59, which included three synonymous mutations. Comparisons of the β-galactosidase assays were performed at various time points of growth to assess the promoter strengths. There was a 30% difference at the late log and stationary phases between the two promoters with the highest activities (type E from SJ24 and the 43895 promoter) and the rest of the promoters (Figure 5). However, it is difficult to derive relationships between *csgD* promoter strengths and biofilm-forming-capabilities when many of the strains in the study carried mutations that limited biofilm formation. Both SJ24 and 43895 strains carried *mlrA* insertions and failed to form biofilms. Promoter types B-D were each represented by strains capable of biofilm formation and biofilm-deficient strains with defects in *mlrA*, *rpoS*, or motility. It is also important to consider that each promoter type may be optimized for, or function differently in, its own respective background. With that limitation recognized, these results indicate that *csgD* promoter sequence variations play a relatively small role in determining promoter strength.

## Discussion

In the previous study of *E. coli* serotype O157:H7 strains, we determined that prophage insertions in *mlrA* and heterogeneous mutations in *rpoS* constituted major obstacles limiting biofilm formation and the expression of extracellular curli fibers under a defined set of conditions. In this study we used similar conditions to investigate the regulation of biofilm formation in a small but diverse collection of non-serotype O157:H7 STEC isolates. The relative ease with which we identified both biofilm-forming and non-biofilm-forming strains in most serotypes suggested that the non-serotype O157:H7 STEC may not encounter the same regulatory restrictions as the O157:H7 strains. Consistent with that observation, we found only 4/19 strains (21%) with prophage insertions in *mlrA*, in contrast to serotype O157:H7 where 53/55 (96.4%) strains carried the prophage [19]. There were two other reports that surveyed the status of *mlrA* (*yehV*) in non-O157 STEC strains, both with a limited number of strains for each serotype [20,21]. One study found no *mlrA* prophage insertion in 15 strains of 11 different non-O157:H7 STEC serotypes [20], all of those serotypes were different from this study. In a different report, the incidence of prophage insertion in *mlrA* was 28% among approx. 70 strains from 42 non-O157 STEC serotypes, compared to 98% for the serotype O157:H7 strains [21]. Although only 1 of the 7 serogroups (O113) reported here was represented in that study [21], their results also suggest that prophage insertions in *mlrA* occur at a much higher frequency in serotype O157:H7 than in many other STEC serogroups. More studies with larger sample sizes are needed to confirm strain, serotype, and serogroup insertion frequencies. 

RpoS mutations were also found in 26% (5/19) of the strains in this study and likely contributed to the biofilm deficiencies. However, assumptions concerning the frequency of *rpoS* mutations and their consequences regarding biofilm formation in comparison with serotype O157:H7 would not be appropriate here since the sampling is limited and might be biased. In a study of RpoS mutations in strains from different sources (soil, food, bovine, or clinical), mutation frequencies in RpoS varied greatly depending on the environment from which strains were collected [31]. Such trends may also apply to non-O157:H7 strains. 

 All 4 strains carrying prophage in *mlrA* possessed wild-type *rpoS* genes, two (DA-33 and SJ15) were successfully complemented for CR binding and curli production by pUC19::*mlrA* but the two O145 strains were not. As the curli structural genes were intact, the O145 strains may carry mutations elsewhere in the regulatory cascade. On the other hand, attempts to complement the CR-binding phenotype in three *rpoS* mutant strains were mostly unsuccessful, even though the catalase activity was restored in these strains. As expected, the two *rpoS* mutant strains SJ29 and DEC10, which showed no catalase activity, were unable to be complemented by pUC19::*mlrA*, indicating that defects in these *rpoS* mutants cannot be overcome by over-producing MlrA on a multi-copy plasmid, and that the expression of *mlrA* on pUC19::*mlrA* was still regulated by RpoS in the same manner as that of the chromosomal copy. On the other hand, CR binding was moderately complemented by pUC19::*mlrA* in strains SJ7 and SJ16; both strains showed low levels of catalase activity and probably produced an attenuated RpoS that was made more effective by increasing the level of MlrA. It seems unorthodox that CR binding in those strains was moderately enhanced by extra copies of the *mlrA* gene, but not so much by adding functional copies of the *rpoS* gene. Since genetic background and the regulation of biofilm-related genes in the non-O157 STEC serotypes may be different from those of the O157:H7 strains, it would be important to conduct genomic comparisons of these strains to gain a more comprehensive understanding of the molecular mechanism involved. We are currently planning to pursue transcriptome analyses of these strains.

Some strains with an intact *mlrA* within each serotype failed to express curli or form biofilm under the conditions used in this study. In the previous study of O157:H7 strains, none of the 55 strains formed biofilm (CV OD_590_ <0.13) and that deficiency could be explained by either prophage insertions or RpoS mutations in all but 1 strain (PA6) [19]. In this study of non-serotype O157:H7 strains, 14 strains showed weak or no biofilm formation (CV OD_590_ <0.29). Nine of those 14 strains (64.3% of the weak biofilm-formers, 47.4% of total) had either prophage insertions in *mlrA* or deficiencies in *rpoS*. Thus 5 of the 19 non-serotype O157:H7 strains (26.3%) had mutations in the biofilm regulatory pathway other than *rpoS* or *mlrA*. In all of those 5 strains, there was complete or nearly complete loss of motility. Flagella have been shown to be essential in the early stages of *E. coli* biofilm development and strains that lacked or produced paralyzed flagella formed only small, dense clusters of attached cells [32]. If lack of flagella-driven motility did compromise biofilm formation in those 5 strains, it would be expected that curli expression, which occurs at a later stage, would remain unaffected as both *mlrA* and *rpoS* were intact. However, while curli was strongly expressed in three of the strains with weak or no motility (FCL1, SJ14, and SJ13), it was present at low levels or absent in strains 98-8338 and 03-4064. Plasmid pUC19::*mlrA*-transformed strains SJ24 and E59 represent additional strains with functional copies of *mlrA* and *rpoS* that are unable to form curli; both showed no motility. While the failure to produce curli could be due to mutations in one of the many other *csgD* regulators, it raises the possibility that certain defects in motility could also have negative effects on curli expression and thus affecting biofilm formation by both inhibiting early motility and then blocking later curli expression. Recent studies have identified factors that regulate both the flagella and *csgD* operons to orchestrate the switch from a motile to a non-motile state [33]. Additional studies are needed to determine if the lack of curli production in these non-motile strains is caused by deficiencies in a common CsgD/flagella regulator.

Prophage insertions in *mlrA*, RpoS mutations, and loss of motility provided reasonable explanations for the biofilm and curli deficiencies of the strains in this study; however, the failure to complement the DEC10 *rpoS* mutation (DEC10 is motile and has an intact *mlrA*) suggests an additional regulatory mutation in that strain. Mutations in any of the many *csgD* regulators would be expected to have detrimental effects on curli expression and biofilm formation but they would likely remain phenotypically silent in the face of the widespread nature of the 3 conditions identified in this study. As such, large-scale comparative genomic sequencing of STEC strains will be needed to identify additional mutations of individual *csgD* regulators. It is conceivable that variations in the *csgD* promoter region where many of these factors bind could cause strain as well as serotype differences in *csgD*/curli expression. Sequencing of the *csgD* promoters in this study indicated that there were conserved differences among and between serogroups. We also tested promoter strengths from each promoter type in a common K12 strain background. Our results indicated that there were serotype differences in the strength of the *csgD* promoters but these differences may not play an important role in CR-affinity or curli production. Additional studies in each of the STEC backgrounds and with integrated single-copy-fusions will be needed to fully test the consequences of these promoter differences.

All biofilm-deficient strains in this study could be characterized as having either: 1) prophage insertion in *mlrA*; 2) *rpoS* mutation; or 3) impaired motility. There was little overlap in these three groups. Of the 14 strains in this study that possessed at least one of the three impairments, none carried both *mlrA* and *rpoS* defects, and only two O145 strains (SJ24 and E59) were non-motile with *mlrA* prophage insertions. In contrast, 38 of the 53 serotype O157:H7 strains (71.7%) had altered RpoS in addition to prophage insertions in *mlrA*, suggesting that the accumulation of these two genotypes were not mutually exclusive. The only strains in the present study that had prophage insertions and one other condition (lack of motility) were the two O145 strains, which may indicate that there was selection pressure against accumulating multiple defects in the biofilm pathways in most non-O157:H7 STEC strains. Interestingly, O145 strains had repeatedly grouped closely with serotype O157:H7, the most successful pathogenic serogroup among the STEC, either by sequence similarity, promoter strength, or other phenomena examined in this study. These results agreed with the genome comparisons showing that O145 and O157 were closely related (X. Yan, personal communication of unpublished results). 

In this study we tested a small collection of 19 non-serotype O157:H7 STEC strains from the 7 important O-serogroups (O26, O45, O103, O111, O113, O121, and O145) to evaluate whether prophage insertions in *mlrA* and mutations in *rpoS* present major barriers to curli expression and biofilm formation as they do in strains of serotype O157:H7. We discovered that, while both genotypes are encountered, *mlrA* prophage insertions are unlikely to be as common as they are in serotype O157:H7. We also discovered that deficiencies in motility form a third general condition that, along with prophage insertions and *rpoS* mutations, provide a plausible explanation for the lack of biofilm formation noted in all biofilm-deficient strains in this study. While mutations in other *csgD* regulators, as observed for strain DEC10, await further studies, we have described conserved serotype variations in the *csgD* promoter that add to the regulatory complexity of curli and biofilm regulation in the STEC strains. 

## Supporting Information

Figure S1 **PCR characterization of *stx_1_*, *stx_2_*, and *mlrA* genes.** (A) *stx_1_*, (B/C) *stx*_*2*_, and (D) *mlrA* multiplex PCR. Ladder: 1kb Extension ladders (A-C) or 100-bp DNA ladder (D).
(TIFF)
