# Supplementary figures and images for: Phenotypic and Genotypic Characterization of Biofilm Forming Capabilities in Non-O157 Shiga Toxin-Producing Escherichia coli Strains
Source: PLoS One. 2013 Dec 27;8(12):e84863. doi: 10.1371/journal.pone.0084863 (PMC3874044; doi:10.1371/journal.pone.0084863)

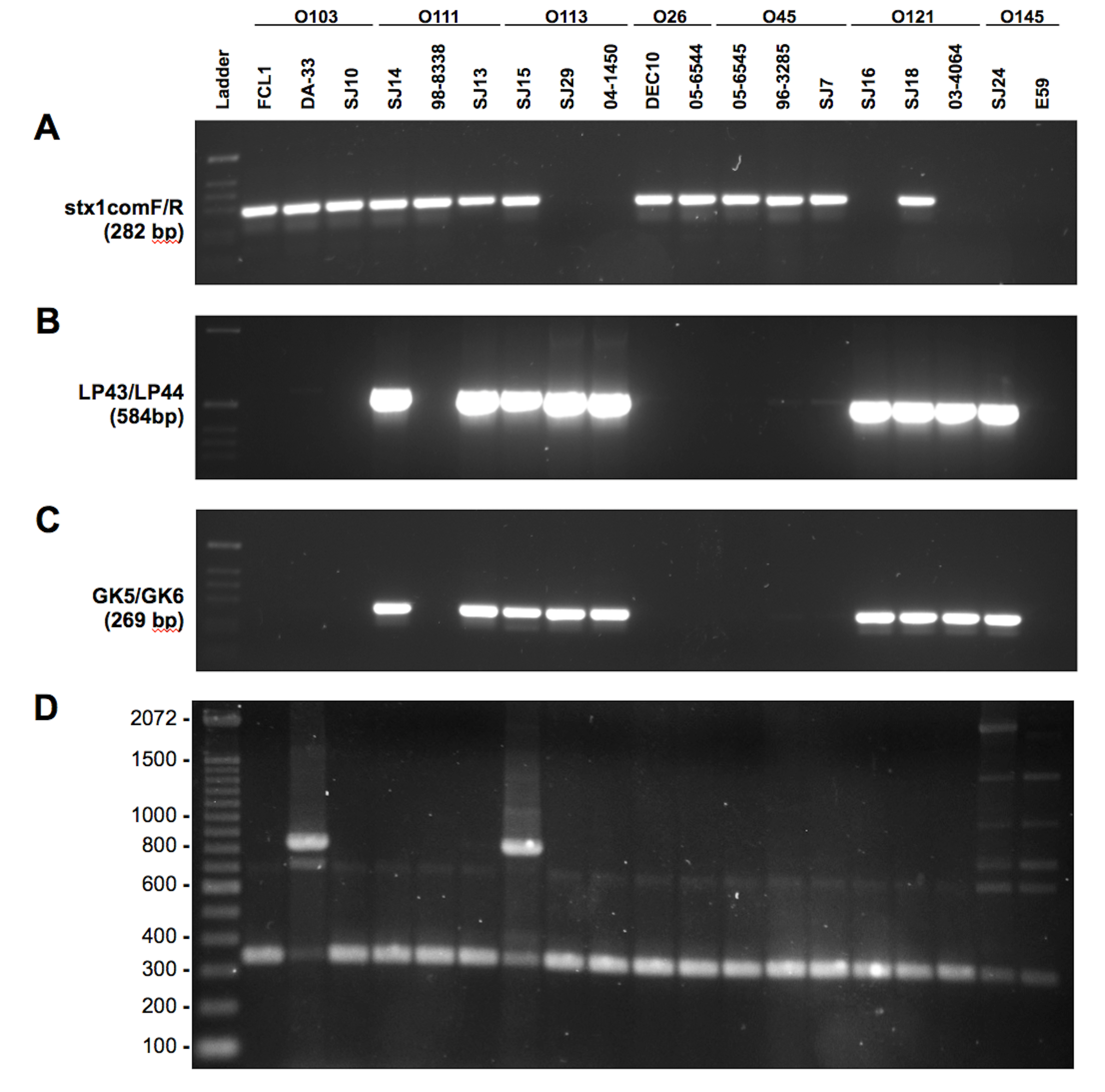

Supplement: Figure S1 — PCR characterization of stx1, stx2, and mlrA genes. (A) stx1, (B/C) stx 2, and (D) mlrA multiplex PCR. Ladder: 1kb Extension ladders (A-C) or 100-bp DNA ladder (D). (TIFF) [file pone.0084863.s001.tiff]
